# Supplementary material for: Somatic Mutations in Exon 7 of the TP53 Gene in Index Colorectal Lesions Are Associated with the Early Occurrence of Metachronous Adenoma
Source: Cancers (Basel). 2022 Jun 7;14(12):2823. doi: 10.3390/cancers14122823 (PMC9221022; doi:10.3390/cancers14122823)
Supplement: Supplementary file 1 [file cancers-14-02823-s001.zip › cancers-1710652-supplementary.pdf]

cancers

MDPI

Supplementary

Somatic Mutations in Exon 7 of the *TP53* Gene in Index Colorectal Lesions Are Associated with the Early Occurrence of Metachronous Adenoma

Tereza Hálková [1], Renata Ptáčková [1], Anastasiya Semyakina [1], Štěpán Suchánek [2,3,*], Eva Traboulsi [4], Ondřej Ngo [5], Kateřina Hejcmanová [5], Ondřej Májek [5], Jan Bureš [3], Miroslav Zavoral [2,3], Marek Minárik [1,6,7] and Lucie Benešová [1]

Table S1. Mutations found in index lesions and the occurrence of early metachronous adenoma.

Patient ID	Mutation in index lesions	Synchronous lesion	Early occurence of metachronous adenoma
1	KRAS	No	non-advanced adenoma
2	No mutation	No	non-advanced adenoma
3	KRAS	Yes	non-advanced adenoma
5	KRAS, APC	No	non-advanced adenoma
6	KRAS, APC	No	normal
7	No mutation	Yes	normal
11	BRAF, TP53-7, APC	Yes	non-advanced adenoma
13	KRAS, TP53-5, APC	Yes	normal
15	KRAS, TP53-8	No	normal
16	BRAF	Yes	hyperplastic polyp
18	BRAF	No	normal
22	KRAS, PIK3CA9	Yes	normal
23	KRAS, PIK3CA9, TP53-6	No	normal
25	KRAS, TP53-5	No	non-advanced adenoma
28	No mutation	No	normal
31	APC	Yes	hyperplastic polyp
32	No mutation	No	normal
33	KRAS	Yes	non-advanced adenoma
34	KRAS, TP53-8, APC	No	normal
35	KRAS, BRAF	Yes	hyperplastic polyp
36	BRAF	No	normal
37	KRAS	Yes	normal
38	KRAS, TP53-8	No	non-advanced adenoma
39	BRAF, TP53-5, TP53-7, TP53-8, APC	Yes	non-advanced adenoma
40	KRAS	Yes	non-advanced adenoma
41	KRAS	No	normal
43	KRAS, BRAF, APC	No	normal
48	KRAS, TP53-5	Yes	advanced adenoma
52	BRAF, TP53-7	Yes	non-advanced adenoma
53	KRAS	No	non-advanced adenoma
55	KRAS, APC	No	non-advanced adenoma
56	KRAS, TP53-5, APC	No	normal
58	TP53-7	Yes	advanced adenoma
59	No mutation	Yes	non-advanced adenoma
64	APC	No	advanced adenoma
66	No mutation	Yes	normal
67	KRAS, TP53-6	No	normal
68	No mutation	Yes	non-advanced adenoma
73	BRAF	No	hyperplastic polyp
74	BRAF, TP53-5, TP53-7, TP53-8	Yes	non-advanced adenoma
76	KRAS	Yes	normal
77	No mutation	No	hyperplastic polyp
78	KRAS, BRAF, TP53-5, TP53-8	Yes	hyperplastic polyp
83	No mutation	No	normal
84	KRAS	Yes	normal
85	KRAS	Yes	non-advanced adenoma
86	KRAS, TP53-5, TP53-7, TP53-8	Yes	advanced adenoma
87	KRAS, APC	Yes	non-advanced adenoma
88	KRAS, BRAF	No	normal
90	KRAS, TP53-6	Yes	non-advanced adenoma
91	KRAS, TP53-6, APC	No	non-advanced adenoma
92	No mutation	Yes	hyperplastic polyp
93	BRAF	Yes	normal

94	KRAS, BRAF, PIK3CA9, TP53-6, APC	No	normal
95	KRAS, APC	Yes	normal
96	APC	No	advanced adenoma
97	KRAS	No	non-advanced adenoma
98	BRAF	Yes	normal
99	KRAS	Yes	normal
102	No mutation	No	normal
103	No mutation	No	normal
104	No mutation	Yes	advanced adenoma
111	BRAF	No	non-advanced adenoma
112	BRAF	Yes	non-advanced adenoma
113	APC	No	normal
114	BRAF	Yes	non-advanced adenoma
115	TP53-6, TP53-8, APC	Yes	non-advanced adenoma
117	KRAS, APC double mut	No	normal
119	KRAS, APC	No	normal
120	KRAS, APC	No	non-advanced adenoma
122	No mutation	Yes	non-advanced adenoma
125	BRAF	No	hyperplastic polyp
126	TP53-7, TP53-8, APC	Yes	non-advanced adenoma
128	No mutation	Yes	non-advanced adenoma
133	No mutation	No	normal
134	TP53-7	Yes	non-advanced adenoma
135	TP53-8	No	normal
137	No mutation	No	hyperplastic polyp
138	KRAS	No	non-advanced adenoma
139	BRAF	Yes	non-advanced adenoma
140	No mutation	Yes	non-advanced adenoma
143	KRAS	No	advanced adenoma
152	APC	No	normal
